# Co-up-regulation of three P450 genes in response to permethrin exposure in permethrin resistant house flies, *Musca domestica*

**DOI:** 10.1186/1472-6793-8-18

**Published:** 2008-09-25

**Authors:** Fang Zhu, Ting Li, Lee Zhang, Nannan Liu

**Affiliations:** 1 Department of Entomology and Plant Pathology, Auburn University, Auburn, AL 36849, USA; 2 Department of Entomology, University of Kentucky, Lexington, KY 40546, USA; 3 Genomics and Sequencing Laboratory, Auburn University, Auburn, AL 36849, USA

## Abstract

**Background:**

Insects may use various biochemical pathways to enable them to tolerate the lethal action of insecticides. For example, increased cytochrome P450 detoxification is known to play an important role in many insect species. Both constitutively increased expression (overexpression) and induction of P450s are thought to be responsible for increased levels of detoxification of insecticides. However, unlike constitutively overexpressed P450 genes, whose expression association with insecticide resistance has been extensively studied, the induction of P450s is less well characterized in insecticide resistance. The current study focuses on the characterization of individual P450 genes that are induced in response to permethrin treatment in permethrin resistant house flies.

**Results:**

The expression of 3 P450 genes, *CYP4D4v2*, *CYP4G2*, and *CYP6A38*, was co-up-regulated by permethrin treatment in permethrin resistant ALHF house flies in a time and dose-dependent manner. Comparison of the deduced protein sequences of these three P450s from resistant ALHF and susceptible aabys and CS house flies revealed identical protein sequences. Genetic linkage analysis located *CYP4D4v2 *and *CYP6A38 *on autosome 5, corresponding to the linkage of P450-mediated resistance in ALHF, whereas *CYP4G2 *was located on autosome 3, where the major insecticide resistance factor(s) for ALHF had been mapped but no P450 genes reported prior to this study.

**Conclusion:**

Our study provides the first direct evidence that multiple P450 genes are co-up-regulated in permethrin resistant house flies through the induction mechanism, which increases overall expression levels of P450 genes in resistant house flies. Taken together with the significant induction of *CYP4D4v2*, *CYP4G2*, and *CYP6A38 *expression by permethrin only in permethrin resistant house flies and the correlation of the linkage of the genes with resistance and/or P450-mediated resistance in resistant ALHF house flies, this study sheds new light on the functional importance of P450 genes in response to insecticide treatment, detoxification of insecticides, the adaptation of insects to their environment, and the evolution of insecticide resistance.

## Background

Cytochrome P450s constitute the largest gene superfamily and are found in the organs and tissues of many organisms, including mammals, fish, plants, arthropods, fungi, and bacteria. Cytochrome P450s have long been of particular interest because they are critical for the detoxification and/or activation of xenobiotics such as drugs, pesticides, plant toxins, chemical carcinogens and mutagens; and for metabolizing endogenous compounds such as hormones, fatty acids, and steroids. Basal and up-regulation of P450 gene expression can significant affect disposition of xenobiotics or endogenous compounds in the tissues of organisms and thus alter their pharmacological/toxicological effects [1]. Insect cytochrome P450s are known to play an important role in detoxifying insecticides [2,3] and plant toxins [4,5], resulting in the development of resistance to insecticides [3,6-10] and facilitating the adaptation of insects to their plant hosts [11,12]. A significant characteristic of insect P450s that is associated with enhanced metabolic detoxification of insecticides in insects is the constitutively increased levels of P450 proteins and P450 activity that result from constitutively transcriptional overexpression of P450 genes in insecticide resistant insects [3,6-9,13,14]. Another feature of insect P450 genes is that the expression of some P450 genes can be induced by exogenous and endogenous compounds [3], a phenomenon known as induction. It has been suggested that the induction of P450s and their activities in insects is involved in the adaptation of insects to their environment and the development of insecticide resistance [15,16].

While all insects probably possess some capacity to detoxify insecticides and xenobiotics, the degree to which they can metabolize and detoxify these toxic chemicals is of considerable importance to their survival in a chemically unfriendly environment [16] and to the development of resistance. The constitutively increased expression and induction of P450s are thought to be responsible for increased levels of detoxification of insecticides, but unlike the constitutively overexpressed P450 genes, whose association with insecticide resistance has been extensively studied, the induction of P450s by xenobiotics, especially phenobarbital, is less well linked to insecticide resistance, although it is well documented in insects [17-22]. It has been proposed that many chemical inducers act as substrates for P450s and that the induction or modulation of P450s by the substrates will, in turn, reduce the effects of the substrates by enhancing substrate metabolism [23]. It has been suggested that the modulation of gene expression reflects a compromise between the insect's need to both conserve energy and adjust to a rapidly changing environment by enhancing the activity of the detoxification system only when a chemical stimulus occurs [24].

The house fly strain ALHF exhibits high levels of resistance to pyrethroids [25]. Previous research using piperonyl butoxide (PBO), an inhibitor of cytochrome P450s, suggested that P450-mediated detoxification may be one of the major mechanisms involved in the development of pyrethroid resistance in ALHF [25]. Genetic linkage analysis points to the localization of PBO-suppressible-P450-mediated resistance on autosomes 1, 2, and 5 of ALHF [26] and factors on autosome 5 are known to play a major role in P450-mediated resistance. Two P450 cDNAs, *CYP6A36 *and *CYP6A5v2*, have recently been identified as being constitutively overexpressed in ALHF and mapped on autosome 5 [27,28], which is correlated with the linkage of resistance in ALHF. These studies suggest the importance of constitutive overexpression of these two genes in increasing metabolic detoxification of permethrin and in the evolution of permethrin resistance in ALHF. To test whether induction of P450s is also involved in the development of permethrin resistance in ALHF, the current study is focused on the characterization of individual P450 genes from house flies that are induced in response to permethrin challenge in ALHF. Three novel P450 genes, *CYP4D4v2*, *CYP4G2*, and *CYP6A38*, were isolated whose expression was induced by permethrin treatment in ALHF. Genetic linkage studies were then conducted in order to identify a further possible causal link between the expression of these genes in response to insecticide treatment and the development of insecticide resistance in ALHF.

## Results

### Identification of P450 genes in response to permethrin challenge

**Figure 1:**
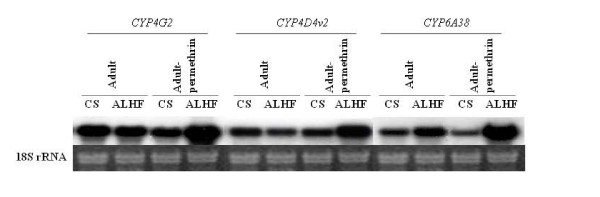
**Northern blot analysis of differentially expressed patterns of *CYP4G2*, *CYP4D4v2*, and *CYP6A38 *between permethrin treated and untreated susceptible CS and resistant ALHF house flies.** mRNAs were isolated from the whole bodies of 20 surviving house flies 24 h after permethrin treatment with 2 *μ*g/fly. Blots were hybridized with the cDNA probes derived from three P450 gene fragments. The ethidium bromide stain of 18S ribosomal RNA in agarose gel is shown at the bottom.

In order to identify the P450 genes raised in response to the insecticide treatment, we used the PCR technique with degenerated PCR primers [29] to amplify P450 cDNAs from house flies. We initially isolated a total of 19 P450 cDNA fragments from ALHF house flies using three primer pairs, C2/Flyh1, C2/Flyc1, and HemeR1/CYP6AD1 (Table 1). Northern blot analysis was conducted using the 19 P450 cDNA fragments as probes to compare expression levels of these cDNAs in both permethrin treated and untreated susceptible CS and resistant ALHF house flies. Three of the 19 P450 cDNAs were found to be significantly induced in the permethrin treated ALHF house flies after 24 hours treatment (Fig. 1) at the pilot dose of LD_50 _that caused ~50% mortality of each house fly strain.

**Table 1:** The primers used for cloning P450 genes, qRT-PCR reactions, and SNP determinations

Gene	Primer Name	Function	Primer Sequence	Position (nt)
*CYP4G2*	AP450HF20F	Full length cloning	5' ATGGATAGTGCAAACAATTCAACTGCG 3'	1 to 27
	SNP450HF20F	SNaP determination	5' AAATGGCCATTTGGTGGCCCAT 3'	174 to 195
	RTP450HF20F	qRT-PCR	5' CGAGGAGGATGATGAAATAAGCAAGC 3'	837 to 862
	RTP450HF20R	qRT-PCR	5' TTGGACATGGCCATCATGGCATCT 3'	957 to 980
	P450HF20R	5'-RACE	5' GCAGGAAGTTGTCACCAAAGATG 3'	1140 to 1162
	P450HF20F	3'-RACE	5' CGTGCATCGCAATCCCCAATAC 3'	1341 to 1362
	AP450HF20R	Full length cloning	5' TTACATAGCTTTCATGGCTTCGGGTC 3'	1625 to 1650

*CYP4D4v2*	AP450HF5F	Full length cloning	5' ATGTTATTTGAATTCCTGGTGGGTC 3'	1 to 25
	SNP450HF5F	SNaP determination	5' ACATGACACCACGACAAGTGG 3'	948 to 968
	RTP450HF5F	qRT-PCR	5' AGGATAAGGAGAAACCGGTGACC 3'	1052 to 1074
	RTP450HF5R	qRT-PCR	5' CAATTGTCGGCACCGATGGATAC 3'	1156 to 1134
	P450HF5R	5'-RACE	5' AGCAACTCAAAATGGCGTACC 3'	1430 to 1410
	AP450HF5R	Full length cloning	5' TAACTACTTGCGAACTCTCAAACCC 3'	1521 to 1497

*CYP6A38*	P450HF17P-F	5' flanking region cloning	5' TGGTCTTCTAGGGGAGAAGACTACCTGC 3'	-676 to -649
	SNP450HF17PF	SNaP determination	5' TTCAGGATTGCTGGGTAGCT 3'	-69 to -50
	P450HF16F-3	Full length cloning	5' CATTATGGAGACTTCGGGAGTTTTG 3'	-4 to 21
	P450HF17P	5' flanking region cloning	5' CGTAGGTTCCTCATGGGGTATACCCAGC 3'	120 to 93
	RTP450HF17F	qRT-PCR	5' CCCTGATGGGCAACATGAATGGAT 3'	122 to 145
	RTP450HF17R	qRT-PCR	5' TAGTTGTTTGTCCAGCAGCACCAC 3'	270 to 247
	P450HF17R	5' RACE	5' CGCTGTACTTCAATAGATTTCCTGC 3'	650 to 626
	P450HF17F	3' RACE	5' TGAGGGTGATACAAAACCAAGC 3'	1017 to 1038
	AP450HF17R	Full length cloning	5' GAGATAATCTCCCACCCCTAAATCG 3'	1520 to 1496

Common	Flyh1		5' GGICCIAGIAACTGCATIGG 3'	
	Flyc1		5' GGAAGTNGACACNTTYATGTT 3'	
	CYP6AD1		5' GTNATHGGHHNBTGYGCHTTYGG 3'	
	HemeR1		5' CCIATGCAGTTICTIGGICC 3'	
	Oligo (dt)		5' TAATACGACTCACTATAGGGAGATTTTTTTTTTTTTTTT 3'	
	C2		5' TAATACGACTCACTATAGGGAGA 3'	
	AP1 (RACE)		5' CCATCCTAATACGACTCACTATAGGGC 3'	
	AP1 (GenomeWalking)		5' GTAATACGACTCACTATAGGGC 3'	
	ActinS1		5' AGGCGAATCGCGAGAAGATG 3'	
	ActinAS1		5' TCAGATCACGACCAGCCAGATC 3'	

**Figure 2:**
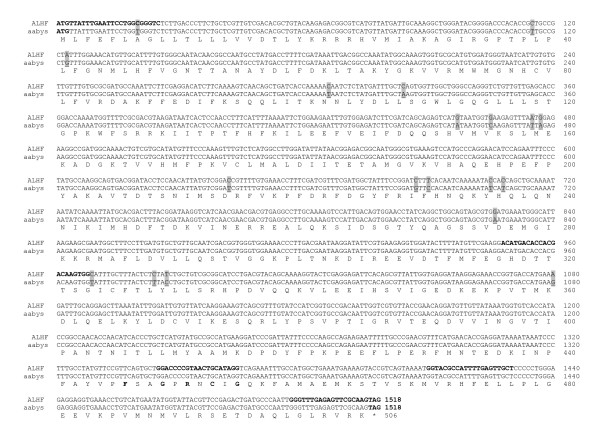
**Alignment of the nucleotide and deduced amino acid sequences of *CYP4D4v2 *in ALHF and aabys house flies.** The nucleotide polymorphisms between ALHF and aabys are highlighted and underlined.

**Figure 3:**
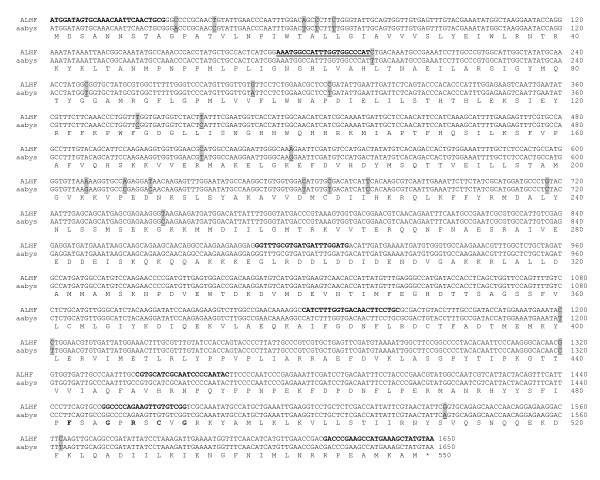
**Alignment of the nucleotide and deduced amino acid sequences of *CYP4G2 *in ALHF and aabys house flies. **The nucleotide polymorphisms between ALHF and aabys are highlighted and underlined.

**Figure 4:**
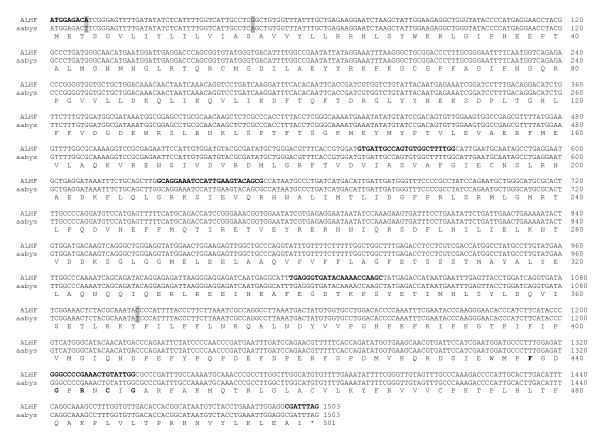
**Alignment of the nucleotide and deduced amino acid sequences of *CYP6A38 *in ALHF and aabys house flies. **The nucleotide polymorphisms between ALHF and aabys are highlighted and underlined.

The full length of the three putative P450 cDNAs were isolated using 5'and/or 3' RACE with the primers (Table 1) designed based on the 5' and/or 3' end sequences of the putative P450 cDNA fragments. The sequences of the 5' and/or 3' RACE amplified cDNAs overlapped with their corresponding putative P450 cDNA fragments. An entire cDNA fragment for each of the putative P450 genes was subsequently amplified for both ALHF and aabys house flies by PCR using the primer pair (Table 1) synthesized based on the respective 5' and 3' end sequences of each gene. The sequences were named *CYP4D4v2 *[accession number: EF615001, ], *CYP4G2 *[accession number: EF615002, ], and *CYP6A38 *[accession number: EF615003, ] by the P450 nomenclature committee (Dr. D. Nelson, personal communication). The cDNA sequences of *CYP4D4v2*, *CYP4G2*, and *CYP6A38 *have open reading frames of 1515, 1647, and 1500 nucleotides encoding proteins of 505, 549, and 500 residues, respectively. Comparison of the deduced protein sequences of CYP4D4v2, CYP4G2, and CYP6A38 between ALHF and aabys revealed identical protein sequences, although several nucleotide polymorphisms were found in the coding regions of these three genes between the two strains (Figs. 2, 3, 4).

### Response of P450 genes to permethrin challenge in resistant and susceptible house flies

**Figure 5:**
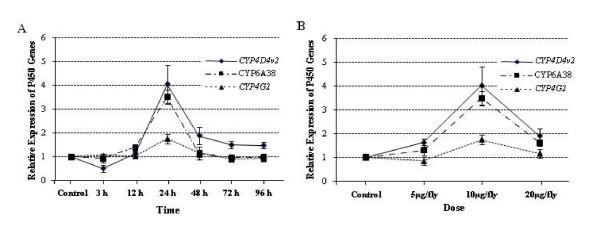
**Time and dose-dependent induction of the expression of three genes was analyzed by qRT-PCR as described in the materials and methods.** A. The duration of the gene expression following permethrin treatment at the dose of LD_50 _(10 *μ*g/fly). B. The expression of the genes 24 h after permethrin treatment with a dose range of LD_10 _(5 *μ*g/fly), LD_50 _(10 *μ*g/fly), and LD_90 _(20 *μ*g/fly). The relative level of gene expression shown in Y axis is the ratio of the gene expression in each treatment in comparison with that in acetone treated flies. The results are shown as the mean ± S.E.

Based on this pilot experiment, we focused our study on *CYP4D4v2*, *CYP4G2*, and *CYP6A38 *in order to further characterize their overexpression in response to permethrin challenge in resistant and susceptible house flies using the quantitative real-time PCR (qRT-PCR) method, as described below under Materials and Methods. To minimize the possibility that these P450 genes have nothing to do with resistance but arose solely because of a strain-strain difference, the study used ALHF and two susceptible house fly strains, CS and aabys. To examine the effect of permethrin on induction of the three target P450s, we measured the expression of the genes in house flies challenged with permethrin at a corresponding dose range (LD_10_, LD_50_, and LD_90_) for various durations. Although no induction was detected in either the susceptible CS flies of the aabys for the dose range and time intervals tested (data not shown), our results showed that permethrin induced all three P450 genes in ALHF with varying levels in a time (24 h)- and dose (LD_50 _-10 *μ*g/fly)-dependent manner (Fig. 5). Based on these data, a dose of 10 *μ*g/fly and a time interval of 24 h were chosen for the further induction studies.

**Figure 6:**
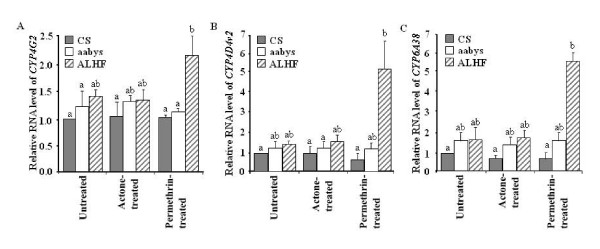
**Induction of *CYP4G2*, *CYP4D4v2*, and *CYP6A38 *in CS and ALHF house flies following treatment with permethrin.** The expression of three genes was analyzed by qRT-PCR as described in the materials and methods. A: Relative expression of *CYP4G2 *in permethrin treated and untreated susceptible CS, aabys and resistant ALHF house flies. B. Relative expression of *CYP4D4v2 *in permethrin treated and untreated CS, aabys and ALHF house flies. C. Relative expression of *CYP6A38 *in permethrin treated and untreated CS, aabys and ALHF house flies. The relative level of gene expression shown in Y axis is the ratio of the gene expression in each strain or each treatment in comparison with that in untreated CS flies. The results are shown as the mean ± S.E. There was no significant difference (*P *≤ 0.05) in the levels of P450 gene expression among the samples with the same alphabetic letter (i.e., a, b, or c).

We detected no significant induction in the expression of the three P450 genes in susceptible CS and aabys house flies that had either been treated with acetone alone or with permethrin solution in acetone compared with untreated house flies (Fig. 6). Similarly, no significant induction was obtained in acetone treated ALHF house flies compared with their untreated counterparts (Fig. 6). However, these three genes were induced at a variety of levels in permethrin treated ALHF house flies compared with untreated or acetone treated ALHF flies; a marked induction of *CYP4D4v2 *and *CYP6A38 *mRNA (~4-fold) were detected in permethrin treated ALHF house flies (Fig. 6B, C), whereas a low level (~1.5-fold) of induction for *CYP4G2 *was detected in the permethrin treated ALHF house flies (Fig. 6A). The significant induction of the three target P450 genes only in ALHF house flies suggests their importance in response to permethrin treatment in the resistant ALHF house flies. No significant differences were observed in the basal expression of *CYP4D4v2*, *CYP4G2*, and *CYP6A38 *between ALHF and both or one of the susceptible CS and aabys strains without permethrin treatment (Fig. 6). These results suggest that, unlike some P450s in which constitutive expression may play important role in insecticide resistance [30-32], *CYP4D4v2*, *CYP6A38*, and/or *CYP4G2*, may be uniquely featured in ALHF in response to the insecticide exposure through induction of their expression, which, in turn, enhances their capacity to detoxify the insecticide and leads to enhanced insecticide resistance.

### Chromosomal linkage and allele determination of *CYP4D4v2*, *CYP4G2*, and *CYP6A38*

**Figure 7:**
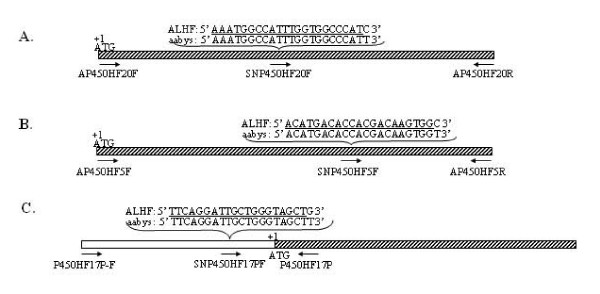
**Graphic representation of *CYP4G2*, *CYP4D4v2*, and *CYP6A38*, showing locations and sequences of SNP-specific primers for SNP determinations and genetic linkage analyses of three P450 genes.** A: *CYP4G2*. B: *CYP4D4v2*. C: *CYP6A38*.

We previously demonstrated that permethrin resistance in ALHF house flies was linked to autosomes 1, 2, 3, and 5, with major factors on autosomes 3 and 5 [26]. We also demonstrated that the P450-mediated resistance in ALHF was predominantly linked to autosome 5 [26]. To determine whether there is a causal link between the P450 genes and insecticide resistance, we examined the genetic linkage of *CYP4D4v2*, *CYP4G2*, and *CYP6A38 *with 5 back-cross (BC_1_) house fly lines derived from crosses of ALHF and a susceptible morphological marker strain, aabys, by allele specific single nucleotide polymorphism (SNP) determination. Sequence comparisons of the three genes between ALHF and aabys revealed several nucleotide polymorphisms in the coding regions of *CYP4D4v2 *and *CYP4G2*, while no nucleotide polymorphisms were identified in the coding region of *CYP6A38*. We therefore cloned a ~700 bp 5' flanking region of *CYP6A38 *in order to genetically map the *CYP6A38 *gene. Comparison of the nucleotide sequence of the 5' flanking region of *CYP6A38 *uncovered several nucleotide polymorphisms between ALHF and aabys (data not shown), so the nucleotide polymorphisms, C to T, C to T, and G to T, in *CYP4D4v2*, *CYP4G2*, and *CYP6A38*, respectively, in ALHF relative to aabys (Fig. 7), were used to determine the linkage of P450 genes relative to the recessive morphological markers in the aabys strain. The SNP determination reactions were conducted for each of the genes using a specific primer (Fig. 7) designed according to the sequences immediately upstream of the nucleotide polymorphism in order to distinguish the single nucleotide polymorphism for the P450 allele in each house fly strain or line. Our results showed that the BC_1 _lines with the genotypes of *ac/ac*, +/*ar*, +/*bwb*, +/*ye*, +/*sw *(A2345), +/*ac*, *ar/ar*, +/*bwb*, +/*ye*, +/*sw *(A1345), +/*ac*, +/*ar*, *bwb/bwb*, +/*ye*, +/*sw *(A1245), and +/*ac*, +/*ar*, +/*bwb*, *ye/ye*, +/*sw *(A1235) were heterozygous for *CYP4D4v2 *and *CYP6A38*, where as the BC_1 _line with the genotype of +/*ac*, +/*ar*, +/*bwb*, +/*ye*, *sw/sw *(A1234) was homozygous for both the *CYP4D4v2 *and *CYP6A38 *alleles from aabys (Table 2). These results strongly indicate that both *CYP4D4v2 *and *CYP6A38 *are located on autosome 5 in house flies. The BC_1 _lines of A2345, A1345, A1234, and A1235 were heterozygous for *CYP4G2*, whereas the A1245 line was homozygous for the *CYP4G2 *allele from aabys (Table 2), indicating that *CYP4G2 *is located on autosome 3 in house flies.

**Table 2:** The nucleotide(s) at the polymorphism site of each P450 gene in different house fly strains and lines generated by the crosses and resistant ALHF and susceptible aabys strains.

		P450 Gene		
		
House Fly Strain		*CYP4G2*	*CYP4D4v2*	*CYP6A38*
ALHF		C	C	G

aabys		T	T	T

BC_1 _Lines* (ALHF ♀x aabys ♂)	A2345	C/T	T/C	G/T
	A1345	C/T	T/C	G/T
	A1245	T	T/C	G/T
	A1235	C/T	T/C	G/T
	A1234	C/T	T	T

BC_1 _Lines* (ALHF ♂x aabys ♀)	A2345	C/T	T/C	G/T
	A1345	C/T	T/C	G/T
	A1245	T	T/C	G/T
	A1235	C/T	T/C	G/T
	A1234	C/T	T	T

* These lines were named according to the autosomes bearing wild-type markers from ALHF. For example, the A1234 strain had wild-type markers on autosomes 1, 2, 3, and 4 from ALHF and the recessive mutant marker on autosome 5 from aabys (see details in Methods).

## Discussion

The primary goal of this study was to investigate whether insecticide resistant insects may be uniquely resistant to insecticides due to their ability to mount an adequate cellular response when challenged with insecticides by up-regulating the production of P450s, which, in turn, may significantly diminish toxicological effects of the insecticides to these insects [1]. It also appears that the induction of gene expression may reflect a good compromise between energy saving (i.e., enhancing the activity of the detoxification system only when a chemical stimulus occurs) and adjustment to a rapidly changing environment [33]. Multiple P450 genes that are induced in insects in response to host plant allelochemicals or secondary products have been extensively studied [3,34-39] and are fairly well documented in terms of their function in the adaptation of insects in "animal-plant warfare" [40] and in the co-evolution of insects and plants [41]. In contrast, P450 gene induction in response to insecticide resistance is less well understood. The current study, therefore, focused on characterization the P450 genes induced in response to a challenge with insecticides in resistant house flies. We restricted this response to permethrin treatment because it is the insecticide that the house flies are resistant to. We found that resistant house flies exposed to permethrin responded by up-regulating a set of P450 genes compared to the levels found in flies that had not been challenged with permethrin, whereas susceptible house flies showed no significant response. This finding indicates that exposure to permethrin induced a response in the expression of multiple P450 genes at different levels in a resistance-specific manner. Similar results have also been reported in *Drosophila melanogaster *[42], where the expression of *CYP6g1 *and *CYP12d1 *were induced in the DDT resistant strains post-exposure to DDT. Our study strongly indicated a clear dose- and time-dependent manner of induction. The lack of induction of P450 gene expression as a result of permethrin treatment at lower doses (LD_10_) in ALHF house flies found in this study is probably due to their rapid metabolism of permethrin at lower doses, which thus never reaches the threshold dose needed for induction [43]. The low or nonexistent levels of induction at higher (LD_90_) doses may indicate a dysfunction of the induction system in insects that have been highly poisoned. A lack of induction of P450s has also been reported in *Drosophila melanogaster *when the insects were challenged with insecticides at concentrations that exceeded LC_99 _[44].

It has been proposed that induction and/or constitutive overexpression of P450s is linked to the adaptation of insects to their environment [15,16]. Further, in many cases increased levels of P450 gene expression have resulted in increased levels of both total P450s and the activities of those P450s, strongly suggesting this as a major cause of insecticide resistance [3,7,10,31,42]. We recently identified two P450 genes that were constitutively overexpressed in ALHF house flies [27,28]. Our current study identified a further three 450 genes that were co-up-regulated in response to permethrin exposure in ALHF house flies. Taken together, these findings suggest both constitutive overexpression and induction mechanisms participate in increasing P450-mediated metabolic detoxification of permethrin in resistant ALHF house and imply the role of these genes in the evolution of insecticide resistance. It has been proposed that there could be similar regulatory mechanisms governing P450 constitutive overexpression and induction, and that both contribute to the development of insecticide resistance [15]. Accordingly, we hypothesize that both induction and constitutive overexpress of P450 genes in the resistant ALHF share an altered regulatory system, which differs from that in the susceptible strains and regulate P450 gene expression in resistant house flies. Further study of the regulation of both constitutively overexpressed [27,28] and permethrin induced P450 genes will allow us to test this hypothesis.

Early studies in our laboratory on permethrin resistance in ALHF house flies led to the identification of permethrin resistance that could be largely suppressed by PBO, an inhibitor of cytochrome P450s [25]. Furthermore, genetic linkage studies had associated permethrin resistance in ALHF to the autosomes 3 and 5, while PBO-suppressible resistance (or P450-mediated resistance) had been mainly tied to autosome 5, with minor factors linked to autosomes 1 and 2 [26]. The genetic linkage between an overexpressed P450 gene or protein and insecticide resistance appears to be an important step in establishing a causal link between a P450 gene and its role in resistance [3,6,32,45-47]. We therefore went on to examine the linkage of the three P450 genes with 5 house fly BC_1 _lines derived from crosses of ALHF and a susceptible morphological marker strain, aabys, using allele specific PCR determination. The results revealed that both *CYP4D4v2 *and *CYP6A38 *were located on autosome 5, whereas *CYP4G2 *was located on autosome 3, of the house flies. Given that *CYP4D4v2 *and *CYP6A38 *are highly induced in ALHF and specifically located on autosome 5, on which P450-mediated resistance in ALHF has been mapped, it seems likely that the induction of *CYP4D4v2 *and *CYP6A38 *plays an important role in the development of insecticide resistance in ALHF house flies. Compared to the induction of the other two P450 genes, the relatively low level (~1.5-fold) of *CYP4G2 *induction obtained in the ALHF house flies may suggest its relatively minor role in resistance.

*CYP4G2 *has been linked on autosome 3. To our knowledge, this is the first report of a P450 gene located on autosome 3 of house flies. An earlier study indicated that although factors on autosome 3 were very important in the overall level of permethrin resistance in ALHF house flies [26], the resistance in ALHF governed by them was not suppressed by PBO [26]. These conflicting results may suggest that an ~1.5-fold level of induction of a P450 gene is too small to be detected by a synergism study. Alternatively, since PBO appears not to be a perfect inhibitor for some of the P450s responsible for resistance [3,48,49], the product of *CYP4G2 *may not be sensitive to the inhibition of PBO and the corresponding level of resistance due to the metabolism ofthe gene may therefore not be suppressed by PBO.

## Conclusion

This study provides direct evidence that multiple P450 genes, *CYP4D4v2*, *CYP4G2*, and *CYP6A38*, are up-regulated in insecticide resistant house flies through the induction mechanism. Taken together with the induction of *CYP4D4v2*, *CYP4G2*, and *CYP6A38 *only in resistant house flies and the correlation linking the genes with the development of resistance and/or P450-mediated resistance in ALHF, this study suggests the functional importance of these three P450 genes in the increased detoxification of insecticides in ALHF. Our previous studies [27,28] have also indicated that two P450 genes are constitutively overexpressed in ALHF house flies. Taken together, these studies indicate that both P450 induction and constitutive overexpression may be co-responsible for detoxification of insecticides, evolutionary insecticide selection, and the ability of insects to adapt to changing environments.

## Methods

### House fly strains

Three house fly strains were used in this study. ALHF is a wild-type strain collected from a poultry farm in Alabama in 1998, selected with permethrin for 6 generations, achieving a 6,600-fold resistance, and maintained under biannual selection with permethrin [25,26]. CS is a wild type insecticide-susceptible strain. aabys is an insecticide-susceptible strain with recessive morphological markers ali-curve (*ac*), aristapedia (*ar*), brown body (*bwb*), yellow eyes (*ye*), and snipped wings (*sw*) on autosomes 1, 2, 3, 4, and 5, respectively. Both CS and aabys were obtained from Dr. J. G. Scott (Cornell University).

### Permethrin challenge experiments

Two-day old resistant ALHF and susceptible CS house flies were treated with permethrin by topical application [25,26] with 0.5 ul permethrin solution (in acetone) and acetone alone dropped on the thoracic notum. Preliminary dose range, time course, and P450 gene induction assays were performed with a corresponding dose range of LD_10_, LD_50_, and LD_90 _(2 ng/fly, 10 ng/fly, and 20 ng/fly for susceptible CS and aabys; 5 *μ*g/fly, 10 *μ*g/fly, and 20 *μ*g/fly for ALHF). Based on the results, in which the induction of P450s reached its peak at 24 h after ALHF were treated with permethrin at the dose of LD_50_, the dose of 20 *u*g/fly that resulted in 50–60% mortality for resistant ALHF were chosen as the challenge dose. The surviving flies were collected for RNA extraction after exposure to permethrin. The control flies that had not been exposed to permethrin treatment and acetone treated flies were collected on the same day as the permethrin treated flies. The experiments were repeated three times.

### RNA extraction, cDNA preparation, and the putative P450 gene isolation

Total RNAs were extracted from the house flies using the acidic guanidine thiocyanate-phenol-chloroform method [7]. mRNA was isolated with oligotex-dT suspension as described by the manufacturer (QIAGEN). The first strand cDNA was synthesized with SuperScript II reverse transcriptase (Stratagene) and an antisense 5'-anchored oligo(dT) primer (Table 1) [50]. The PCR products were amplified using three primer pairs of C2/Flyh1, C2/Flyc1 and HemeR1/CYP6AD1 (Table 1). CYP6AD1 was designed based on a conserved amino acid region found in the house fly, *Drosophila*, and mosquitoP450 sequences after alig nment of these insect P450 genes; Flyh1 was designed based on the heme binding consensus sequence [51]; HemeR1 was generated based on the complementary sequence of Flyh1; and Flyc1 was synthesized based on a conserved 13 amino acid region found in rat, human, and insect P450 sequences [51]. The PCR products were cloned into PCR™ 2.1 Original TA cloning vector (Invitrogen) and sequenced. Cloning and sequence analyses of P450 gene fragmentswere repeated at least three times with different preparations of mRNAs. Three TA clones from each replication were sequenced.

### Rapid amplification of cDNA ends (RACE) of the putative P450 gene fragments

RACE was carried out using the Marathon™ cDNA Amplification Kit (Clontech) as described by the manufacturer and Liu and Zhang [51]. The first strand cDNAs were synthesized with AMV reverse transcriptase using ALHF mRNAs as templates. The double strand cDNA was synthesized following the protocol described by the manufacturer (Clontech). Adaptors were ligated to both ends of each double strand cDNA using T4 DNA ligase as described by the manufacturer. The 5' and/or 3' ends of the P450 cDNA fragmentswere amplified by PCR using adapter primer AP1 and gene specific primers generated based on the 5' and/or 3' end sequences of the putative P450 cDNA fragments. The full lengths of putative P450 cDNAs were subsequently generated by reverse transcription PCR (RT-PCR) using specific primer pairs of AP450HF5F/AP450HF5R, AP450HF20F/AP450HF20R and P450HF16F-3/AP450HF17R (Table 1) synthesized based on the 5'and 3'end sequences of the putative P450 genes. Cloning and sequence analyses of the P450 cDNAs were repeated at least three times and three TA clones from each replication were verified by sequencing.

### Cloning and sequencing of the 5' flanking region of *CYP6A38 *from ALHF and aabys

House fly genomic DNAs were digested with 5 different restriction enzymes using the Universal GenomeWalker™ Kit (Clontech) and generated 5 adaptor-ligated ALHF genomic DNA libraries as described by the manufacture. The adaptor ligated DNA fragments in the GenomeWalker libraries were amplified by PCR with Advantage *Tth *polymerase (Clontech), the antisense primer, P450HF17P (Table 1), based on the 5' coding region of the *CYP6A38*, and a sense primer, AP1, based on the sequence of the adaptor. The PCR products were cloned into the TA cloning vector (Invitrogen)and sequenced. Cloning and sequence analyses of PCR products were repeated at least three times each with three TA clones from each replication. The 5' flanking region of *CYP6A38 *in aabys was subsequently generated by PCR from the genomic DNA using a primer pair, P450HF17P-F/P450HF17P (Table 1) designed according to the 5' flanking region of *CYP6A38 *in ALHF.

### Northern blot analysis

Northern blot analyses were performed according to Sambrook et al. [52]. Twenty micrograms of total RNA from each sample were fractionated on 1% formaldehyde denaturing agarose gel and transferred to Nytran membranes (Schleicher and Schuell) [52]. The P450 cDNAs were labeled with [α-^32^P] dCTP using a Primer-It II Random Primer Labeling kit (Stratagene) and hybridized with RNA blots using QuickHyb solution (Stratagene). The amount of RNA loaded in each lane was standardized by comparing the density of the 18S ribosomal RNA band on agarose gel under UV light before transfer [53]. All Northern blot analyses were repeated three times with different preparations of RNA samples.

### Quantitative Real-time PCR (qRT-PCR)

Total RNA samples (0.5 *μ*g/sample) were reverse-transcribed using SuperScript II reverse transcriptase (Stratagene) in a total volume of 20 *μ*l. The quantity of cDNAs was measured using a spectrophotometer prior to qRT-PCR. qRT-PCR was performed with the SYBR Green master mix Kit and ABI 7500 Real Time PCR system (Applied Biosystems). Each qRT-PCR reaction (25 *μ*l final volume) contained 1× SYBR Green master mix, 1 *μ*l of cDNA, and a gene specific primer pair, RTP450HF5F/RTP450HF5R, RTP450HF20F/RTP450HF20R, or RTP450HF17F/RTP450HF17R (Table 1), at a final concentration of 3–5 *μ*M. A 'no-template' negative control and all samples were performed in triplicate. The reaction cycle consisted of a melting step of 50°C for 2 min then 95°C for 10 min, followed by 40 cycles of 95°C for 15 sec and 60°C for 1 min. Specificity of the PCR reactions was assessed by a melting curve analysis for each PCR reaction using Dissociation Curves software [54]. Relative expression levels for specific genes were calculated by the 2^-ΔΔC(t) ^method using SDS RQ software [55]. The *β*-actin gene, an endogenous control, was used to normalize expression of target genes [56,57]. The preliminary assay had shown that the *β*-actin gene remained constant in different tissues and in both permethrin treated and untreated house flies and could therefore be used for internal normalization in qRT-PCR assays. Each experiment was repeated three times with different preparations of RNA samples. The statistical significance of the gene expressions was calculated using a Student's *t*-test for all 2-sample comparisons and a one-way analysis of variance (ANOVA) for multiple sample comparisons (SAS v9.1 software); a value of *P *≤ 0.05 was considered statistically significant.

### Genetic linkage analysis of cytochrome P450 genes

To determine the genetic linkage of P450 genes, a genetic cross experiment was conducted [26,58]. Briefly, reciprocal crosses between ALHF and aabys were conducted and F_1 _males were back-crossed to aabys females. Five lines were saved from the back-cross generation (BC_1_) with the genotypes of: *ac/ac*, +/*ar*, +/*bwb*, +/*ye*, +/*sw *(A2345); +/*ac*, *ar/ar*, +/*bwb*, +/*ye*, +/*sw *(A1345); +/*ac*, +/*ar*, *bwb/bwb*, +/*ye*, +/*sw *(A1245); +/*ac*, +/*ar*, +/*bwb*, *ye/ye*, +/*sw *(A1235); and +/*ac*, +/*ar*, +/*bwb*, +/*ye*, *sw/sw *(A1234). Since crossing over does not or very rarely occurs in male flies [59], the presence of a mutant phenotype indicated that the respective autosome with a mutant-type marker was derived from the aabys females. The genotype of each line was homozygous for the recessive mutant allele from aabys and heterozygous for the dominant wild-type alleles from ALHF. These lines were named according to the autosomes bearing wild-type markers from ALHF. For example, the A1234 strain had wild-type markers on autosomes 1, 2, 3, and 4 from ALHF and the recessive mutant marker on autosome 5 from aabys.

The P450 specific alleles in ALHF and aabys and five house fly BC_1 _lines were genetically mapped by SNP determination using an ABI Prism SNaPshot Multiplex Kit and analyzed on the ABI Prism^® ^3100 Genetic Analyzer using Genemapper software according to the manufacture's instructions (A&B Applied Biosystems). Briefly, the cDNA fragments, which covered the SNP sites of P450 cDNAs, were generated by PCR. The SNP determination reactions were conducted for *CYP4D4v2*, *CYP4G2*, and *CYP6A38 *using allele specific primers, SNP450HF5F, SNP450HF20F, and SNP450HF17F, respectively (Table 1, Fig. 7), designed according to the sequences immediately upstream of the nucleotide polymorphism to distinguish the single nucleotide polymorphism for the P450 allele in each house fly strain or line. Three replications of the SNP determination were carried out with different preparations of the PCR templates. To confirm that the PCR products used for the SNP determination were in fact the P450 gene fragments, the PCR products were sequenced at least once each.

## Abbreviations

ANOVA: a one-way analysis of variance; BC_1_: back-cross generation 1; PBO: piperonyl butoxide; qRT-PCR: quantitative real-time PCR; RACE: rapid amplification of cDNA ends; RT-PCR: Reverse transcription PCR; SNP: single nucleotide polymorphism.

## Authors' contributions

FZ was involved in coordinating and carrying out the experiments described in this report and wrote the manuscript. TL was involved in the dose range and time course studies. LZ was involved in the review of data, supervising implementation of the overall process, sequencing, and editing the manuscript. NL was involved in the overall design of study, review of data, and editing the manuscript. All authors read and approved the final manuscript.
